# METS-IR and all-cause mortality in Korean over 60 years old: Korean genome and epidemiology study-health examinees (KoGES-HEXA) cohorts

**DOI:** 10.3389/fendo.2024.1346158

**Published:** 2024-03-20

**Authors:** Ha Eun Ryu, Dong Hyuk Jung, Seok-Jae Heo, Byoungjin Park, Yong Jae Lee

**Affiliations:** ^1^ Department of Family Medicine, Yongin Severance Hospital, Yongin-si, Republic of Korea; ^2^ Department of Family Medicine, Yonsei University College of Medicine, Seoul, Republic of Korea; ^3^ Division of Biostatistics, Department of Biomedical Systems Informatics, Yonsei University College of Medicine, Seoul, Republic of Korea; ^4^ Department of Family Medicine, Gangnam Severance Hospital, Seoul, Republic of Korea

**Keywords:** METS-IR, mortality, aging, cardiovascular disease, cancer, insulin resistance

## Abstract

**Background:**

The metabolic score for insulin resistance index (METS-IR) is a novel non insulin-based marker that indicates the risk for metabolic syndrome and type 2 diabetes mellitus (T2DM). However, METS-IR has not been investigated in relation to all–cause mortality. We investigated the longitudinal effect of METS-IR on all–cause mortality in a significantly large cohort of Korean adults over 60 years old.

**Methods:**

Data were assessed from 30,164 Korean participants over 60 years of age from the Korean Genome and Epidemiology Study-Health Examinees (KoGES-HEXA) cohort data, linked with the death certificate database of the National Statistical Office. The participants were grouped into three according to METS-IR tertiles. We used multivariate Cox proportional-hazard regression models to prospectively assess hazard ratios (HRs) for all-cause mortality with 95% confidence intervals (CIs) over an 11-year postbaseline period.

**Results:**

During the mean 11.7 years of follow-up, 2,821 individuals expired. The HRs of mortality for METS-IR tertiles were 1.16 (95% CI, 1.01–1.34) in T3 after adjustment for metabolic parameters, but the T2 did not show statistical significance towards increases for incident mortality respectively. In subgroup analysis depending on the cause of mortality, higher METS-IR was associated with cancer mortality (HR, 1.23, 95% CI, 1.01–1.51) but not with cardiovascular mortality (HR, 1.14, 95% CI, 0.83–1.57) after adjustment for the same confounding variables.

**Conclusion:**

The METS-IR may be a useful predictive marker for all-cause mortality and cancer mortality, but not for cardiovascular mortality in subjects over 60 years of age. This implies that early detection and intervention strategies for metabolic syndrome could potentially benefit this identified group.

## Introduction

Aging is the process characterized by the continual accumulation of changes that result in sequential transformations as one advances in age (1). It stands as the most significant and unalterable factor contributing to the risk of diseases and mortality (2). In recent decades, while advancements in public health have led to reduced mortality rates and increased life expectancy (3), an undeniable challenge has emerged. The notable increase in life expectancy has given rise to a growing population of individuals afflicted by chronic diseases associated with aging (4). Particularly, in today’s reality, where an unhealthy lifestyle accelerates susceptibility to diseases, this trend is gaining momentum (5). As a result, our focus is shifting towards the importance of healthy life expectancy (6). In a world marked by rapid demographic changes and a progressively aging population, the importance of managing the health of the older population has gained unprecedented significance.

Insulin resistance (IR) is defined as a state in which insulin exhibits reduced responsiveness in target tissues, despite its sufficient secretion (7). The unfavorable metabolic changes and disrupted glucose metabolism induced by IR can ultimately lead to the generation of oxidative stress and trigger inflammatory responses that result in cellular damage (8). Consequently, IR is associated with chronic conditions such as metabolic syndrome, hypertension and T2DM, and it extends to the development of serious health issues, including cardiovascular diseases (CVD), cancer, and, in some instances, mortality (9, 10). Globally, the leading causes of mortality are primarily dominated by CVD and cancer (11). In addition, the prevalence of IR is increasing worldwide, driven by factors such as excessive nutrition and sedentary modern lifestyles (12). Within the context of global aging and these prevailing trends, early detection of IR in the elderly is valuable as it enables the evaluation of the risk for major diseases like CVD and cancer, along with their associated mortality, and facilitates preventive management.

The hyperinsulinemic-euglycemic clamp is considered the gold standard for assessing IR (13). However, this method is often impractical for routine IR assessment, leading to the development of several surrogate IR markers to meet this demand (14, 15). Among these markers, the recently introduced novel non-insulin-based IR marker, METS-IR, has been proposed and validated against the hyperinsulinemic-euglycemic clamp, confirming its efficacy (16). METS-IR is a convenient tool for routine health monitoring, utilizing easily assessable metrics such as fasting plasma glucose (FPG), triglyceride (TG), high-density lipoprotein cholesterol (HDL-C), and body mass index (BMI). This simplicity makes it highly accessible and sustainable for primary care in the older population. Additionally, previous studies targeting patients without diabetes revealed that METS-IR outperformed metabolic syndrome in predicting ischemic heart disease, a key age-related ailment (17, 18). In comparison to the metabolic syndrome, acknowledged as a risk factor for major age-associated diseases with insulin resistance as its primary pathophysiology (19), METS-IR demonstrated its utility and excellence as an alternative marker for insulin resistance.

While METS-IR is widely used as an indicator of IR and has been linked to conditions such as hypertension, T2DM, and CVD in previous studies (17, 20–22), there is currently no research, to our knowledge, that has explored the connection between METS-IR and both all-cause and cause-specific mortality in individuals aged 60 and above. Therefore, we prospectively investigated the relationships between METS-IR and all-cause, as well as cause-specific mortality, among individuals aged over 60 years within the Korean population.

## Materials and methods

### Study design and participants

The Health Examinees Cohort (HEXA) is a large, government-funded prospective cohort study to identify genetic and environmental factors for common complex diseases in. The cohort of participants consisted of community dwellers and participants, men and women, aged ≥ 40 years at baseline who were recruited from the National Health Examinee Registry. These participants were recruited during the baseline survey, conducted from 2004 to 2013, at 38 health examination centers and hospitals in the eight regions of South Korea. The participants were then asked to return periodically to complete the follow-up surveys by mail and telephone. Details of the study have been published elsewhere (23). For the analysis of this study, anonymized data of 173,195 participants aged ≥ 40 years were linked with the death certificate database of the National Statistical Office. The data set of those consists of anthropometric and clinical measurements, lifestyle (i.e., diet, smoking, alcohol drinking, and physical activity), and the Food Frequency Questionnaire. In the current study, we included a total of 30,164 participants whose medical history and mortality records were available.

**Figure 1:**
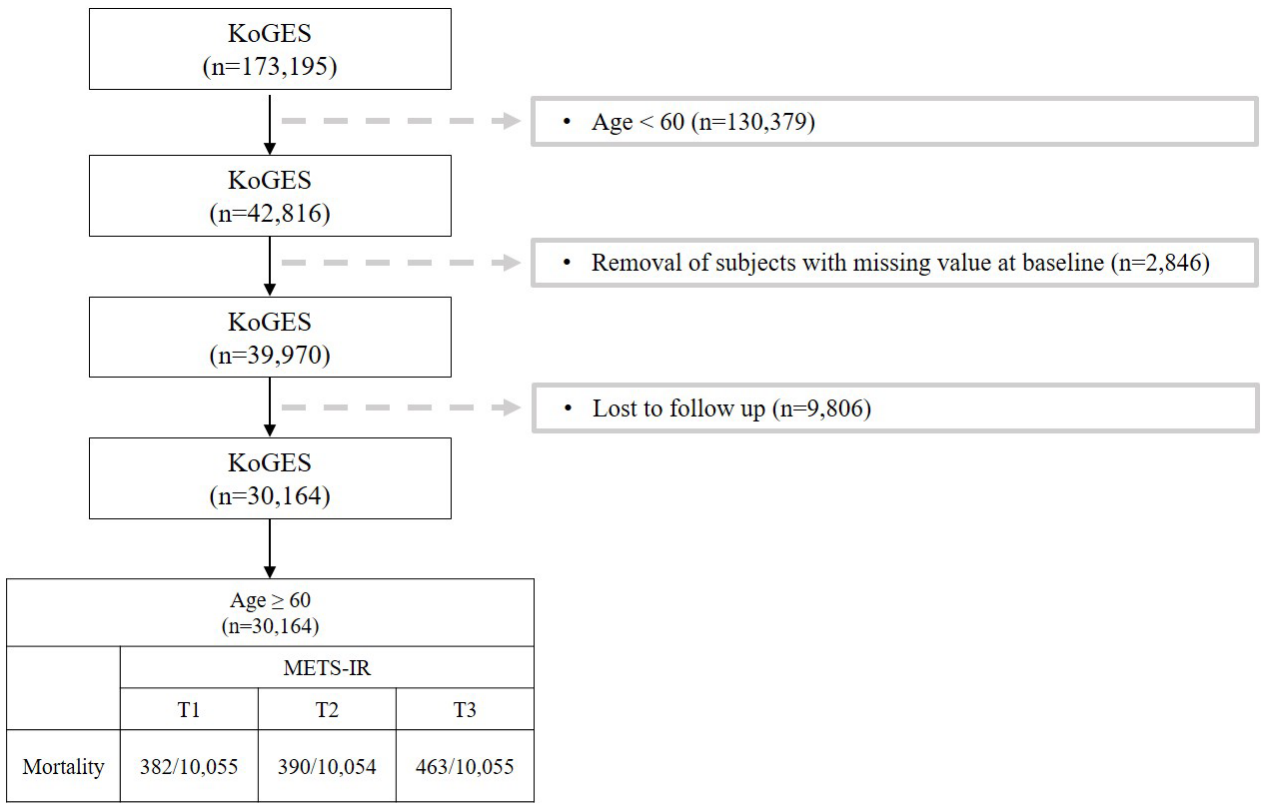
Flowchart for the selection of study participants.

This study investigated the risk factors for metabolic syndrome (METS-IR) in the population over 60 years of age. **Figure 1** shows a flow chart describing the study. Out of a total of 173,195 participants, we excluded 130,379 who were under 60. Additionally, 2,846 participants with missing covariates were excluded, along with another 9,806 due to follow-up loss. Participant follow-up employed both active and passive methods. Active methods involved sending information leaflets by mail and making phone calls, while passive methods identified cases through Korean health-related databases (24). Main reasons for follow-up refusal included changes in contact information, being too busy to attend, and not responding to phone calls (23). Imputation analysis was conducted to address the issue of excessive exclusions due to follow-up loss, confirming no significant bias resulting from missing data (**Supplementary Table S5**). Ultimately, after these exclusions, 30,164 participants remained in the study. This study was conducted under the Institutional Review Board (IRB) approval of Yongin Severance Hospital (IRB number: 9-2023-0018).

### Data collection

Every participant granted informed consent for baseline data and biospecimen collection, and underwent both an interview and physical examination. Ethical approval was secured from the institutional review boards of the National Research Institute of Health and collaborators of the KoGES groups (23). Each participant completed a comprehensive questionnaire that captured information about his or her lifestyle and medical history. Smoking status was divided into never-smoker, ex-smoker, and current smoker. Regular alcohol drinker was defined as consuming more than 140 grams per week, based on the frequency of alcohol consumption reported by the subjects. Body weight and height were measured with an accuracy of 0.1 kg and 0.1 cm, respectively. Participants were instructed to wear light indoor clothing and not to wear shoes during measurement. BMI was calculated by dividing weight divided by height squared (kg/m^2^). Systolic blood pressure (SBP) and diastolic blood pressure (DBP) were assessed by means of a standard mercury sphygmomanometer (Baumanometer, W.A. Baum Co Inc., Copiague, NY, USA) while participants were in a seated and rested for 10 minutes Mean arterial pressure was calculated from the measured SBP and DBP values. Hypertension was defined as an SBP ≥140 mmHg, a DBP ≥90 mmHg, or current use of hypertension medication. Blood samples were collected from the subjects through an antecubital vein after a 12-hour overnight fast. Concentrations of FPG, total cholesterol, TG, HDL-C, aspartate aminotransferase (AST), alanine aminotransferase (ALT) and γ-glutamyltransferase (GGT) were measured enzymatically using a Chemistry Analyzer (Hitachi 7600, Tokyo, Japan up to August 2002 and ADVIA 1650, Siemens, Tarrytown, NY from September 2002). In the HEXA cohort, efforts are made to achieve standardized results through the centralization of sample preparation and management. Each year, on-site inspections, internal and external quality control, inter-laboratory comparisons, measurement traceability evaluations, and trend analysis assessments are consistently conducted for the diagnostic testing institutions. These ongoing checks aim to enhance the reliability of test results (25).

### Assessment of METS-IR

The METS-IR index was computed using the following formulas (16): ln (2 × FPG [mg/dL] + TG [mg/dL]) × BMI (kg/m^2^)/ln (HDL-C [mg/dL]).

### Study outcomes

Mortality status was determined by linking data to the unique personal identification key code system since the HEXA cohort is connected to national data sources that contain mortality records, from the Korea National Statistical Office. Participants were continuously followed from the baseline survey data to the the time of the mortality event, the study end date, or the date of last contact. Participant mortality was monitored from January 2001 to December 2019, with the cause of mortality classified based on the International Classification of Diseases (ICD) codes as listed in the National Mortality Index. All-cause mortality represents all deaths with specified and unknown causes, cancer mortality includes deaths under ICD-10 codes C00-C97, and CVD mortality includes deasths under ICD-10 codes I00-I99.

### Statistical analysis

We categorized the participants into three groups according to the base METS-IR level. The cut-off level of METS-IR is 33.2 and 38.0 in subjects older than 60 years (17, 20, 26–30). All the data presented in this study include means with standard deviations or frequency with percentages. The baseline characteristics of the study population were compared according to METS-IR tertiles using Pearson’s chi-squared test for categorical variables and an analysis of variance (ANOVA) for continuous variables. The primary variables under examination included individual demographic details, anthropometric and biochemical parameters, and lifestyle factors. Kaplan–Meier curves were used to evaluate the cumulative incidence of all-cause mortality, cancer-related and CVD-related mortality. The log-rank test was used to determine whether the distributions of cumulative incidence function for mortality differed among the groups. In multivariable analysis, with the lowest tertiles of all-cause mortality value as the reference group, hazard ratios (HRs) and 95% confidence intervals (CIs) for incident mortality were calculated using the Cox proportional hazards model after adjustment for potential confounding variables. The models included participant characteristics related to mortality in the Korean population as potential confounders (31). For all analyses, R software (version 4.0.2; R Foundation for Statistical Computing, Vienna, Austria) was used. All statistical tests conducted were two-sided, with P values of <0.05 were considered to be statistically significant.

## Results

### Baseline characteristics

During the mean 11.7 years of follow-up period, 2,821 all-cause mortality, 1,235 cancer mortality, 514 CVD mortality, and 1,072 mortality from other reasons occurred among the 30,164 participants. **Table 1** displays the baseline characteristics of the study population according to the METS-IR tertiles. Those in the third METS-IR tertiles had higher levels or proportions of BMI, waist circumference (WC), SBP, TG, FPG, liver enzymes, and lower levels of HDL-C. The third tertile of the METS-IR in population also had the highest proportion of current smoker, hypertension, and T2DM.

**Table 1:** Baseline characteristics of the study population according to the METS-IR tertiles in individuals older than 60 years.

Characteristic	Total	Group 1	Group 2	Group 3	*p* value
	(n=30,164)	T1 [16.7, 33.2] (n = 10,055)	T2 (33.2, 38.0] (n = 10,054)	T3 (38.0, 77] (n = 10,055)	
Sex (men)	12,837 (42.6)	3,948 (39.3)	4,225 (42)	4,664 (46.4)	<0.001
Age (years)	64.3 ± 3.3	64.2 ± 3.3	64.3 ± 3.3	64.4 ± 3.3	0.014
Body mass index (kg/m^2^)	24.3 ± 2.8	21.7 ± 1.8	24.3 ± 1.5	26.9 ± 2.3	<0.001
Waist circumference (cm)	83.4 ± 8.1	77.3 ± 6.5	83.6 ± 5.8	89.4 ± 6.8	<0.001
Systolic blood pressure	127.1 ± 15.1	124.1 ± 15.1	127.4 ± 14.8	129.7 ± 14.9	<0.001
Diastolic blood pressure (mmHg)	77.2 ± 9.4	75.6 ± 9.3	77.3 ± 9.3	78.6 ± 9.3	<0.001
LDL-cholesterol (mg/dl)	119.0 ± 31.1	116.0 ± 29.6	122.7 ± 31.6	118.4 ± 32.7	<0.001
HDL-cholesterol (mg/dl)	51.8 ± 12.4	60.6 ± 12.3	50.9 ± 9.5	43.9 ± 8.9	<0.001
Triglyceride (mg/dl)	126.6 ± 64.9	91.1 ± 40.2	123.0 ± 45.5	165.9 ± 72.2	<0.001
Fasting plasma glucose (mg/dl)	98.7 ± 23.1	93.2 ± 16.2	98.3 ± 22.0	104.7 ± 28.1	<0.001
ALT	22.6 ± 17.7	19.9 ± 19.1	22.1 ± 14.4	25.8 ± 18.7	<0.001
AST	24.8 ± 14.4	24.6 ± 18.5	24.3 ± 11.3	25.6 ± 12.2	<0.001
Creatinine	0.9 ± 0.3	0.8 ± 0.2	0.8 ± 0.2	0.9 ± 0.3	<0.001
Smoking status, n (%)					<0.001
Never smoker	21,005 (69.6)	7,333 (72.9)	7,052 (70.1)	6,620 (65.8)	
Former smoker	6,364 (21.1)	1,821 (18.1)	2,165 (21.5)	2,378 (23.6)	
Current smoker	2,795 (9.3)	901 (9)	837 (8.3)	1,057 (10.5)	
Alcohol intake, n (%)					<0.001
Never drinker	17,556 (58.2)	5,920 (58.9)	5,822 (57.9)	5,814 (57.8)	
Former drinker	1,658 (5.5)	483 (4.8)	541 (5.4)	634 (6.3)	
Current drinker	10,950 (36.3)	3,652 (36.3)	3,691 (36.7)	3,607 (35.9)	
Regular exercise (Yes)	1,6282 (54)	5,501 (54.7)	5,668 (56.4)	5,113 (50.9)	<0.001
Hypertension, n (%)	10,967 (36.4)	2,572 (25.6)	3,645 (36.3)	4,750 (47.2)	<0.001
Diabetes, n (%)	3,928 (13)	775 (7.7)	1,246 (12.4)	1,907 (19.0)	<0.001

P-values were calculated using 1-way ANOVA or Pearson’s chi-square test.

### Hazard ratios for all-cause mortality

**Figure 2:**
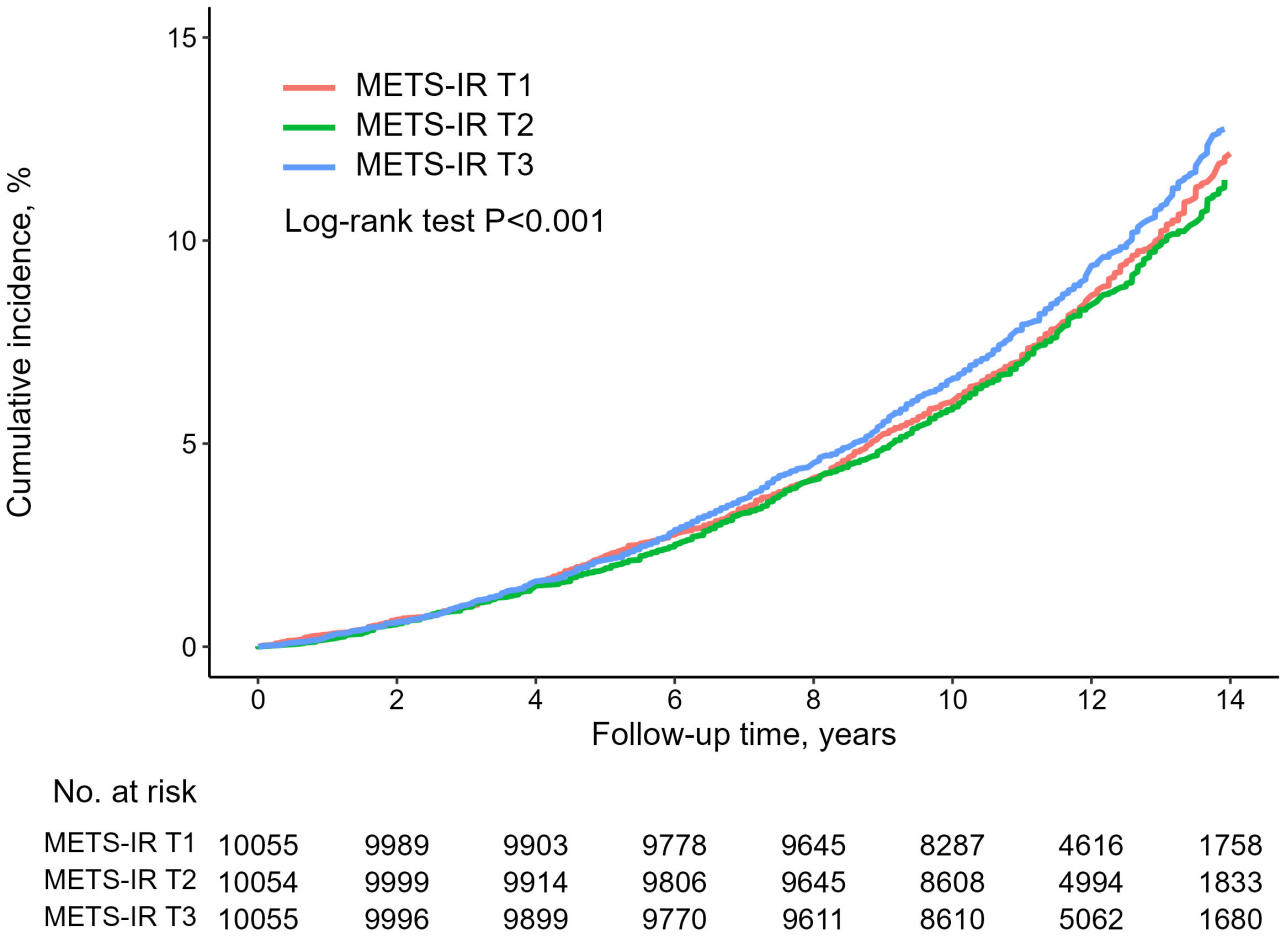
Kaplan-Meier curve for all-cause mortality.

**Table 2** summarizes the association between baseline METS-IR and all-cause mortality within the study population. In the unadjusted model, there was no association with higher all-cause mortality for the highest METS-IR (all-cause mortality: METS-IR [16.7, 33.2] vs METS-IR [38.0, 77], HR, 1.05; 95% CI, 0.96–1.15). However, In the fully adjusted model, contorolling for age, sex, WC, SBP, DBP, creatinine, low-density lipoprotein (LDL) cholesterol, smoking status, alcohol intake, exercise, liver enzymes, hypertension and T2DM, it was found that the highest METS-IR was significantly associated with all-cause (all-cause mortality: METS-IR [16.7, 33.2] vs METS-IR [38.0, 77], HR, 1.16; 95% CI, 1.01–1.34). Additionally, as depicted in **Figure 2**, the Kaplan-Meier survival curve, when stratified by METS-IR tertiles, illustrated an elevation in the cumulative incidence of all-cause mortality corresponding to an increase in METS-IR (log-rank test, P < 0.001).

**Table 2:** Hazard ratios and 95% confidence intervals for All-cause mortality according to METS-IR tertiles in subjects older than 60 years.

		Group 1	Group 2	Group 3	*p* for trend
		T1 [16.7, 33.2] (n = 10,055)	T2 [33.2, 38.0] (n = 10,054)	T3 [38.0, 77] (n = 10,055)	
New cases of death, n		923	905	993	
Mean follow-up, years		11.59	11.75	11.73	
Pearson-years of follow-up		116,497	118,172	117,943	
Incidence rate/1000 person -years		7.92	7.66	8.42	
Model 1	HR (95% CI)	1.00 (reference)	0.95 (0.87-1.05)	1.05 (0.96-1.15)	0.240
	*p* value	–	0.325	0.250	
Model 2	HR (95% CI)	1.00 (reference)	1.11 (1.00-1.23)	1.40 (1.22-1.60)	<0.001
	*p* value	–	0.054	<0.001	
Model 3	HR (95% CI)	1.00 (reference)	1.10 (0.99-1.22)	1.33 (1.16-1.53)	<0.001
	*p* value	–	0.083	<0.001	
Model 4	HR (95% CI)	1.00 (reference)	1.05 (0.95-1.17)	1.16 (1.01-1.34)	0.030
	*p* value	–	0.354	0.030	

Model 1: Unadjusted.

Model 2: adjusted for age, sex and WC.

Model 3: adjusted for age, sex, WC, SBP, DBP, ALT, AST, creatinine and LDL.

Model 4: adjusted for age, sex, WC, SBP, DBP, ALT, AST, creatinine, LDL, smoke, drink, exercise, HTN and DM.

### Subgroup analysis for incident mortality

In the subgroup analysis depending on the cause of death, higher METS-IR was associated with cancer mortality but this association was not observed in CVD mortality (**Table 3**).

(cancer mortality: METS-IR [16.7, 33.2] vs METS-IR [38.0, 77], HR, 1.23; 95% CI, 1.01–1.51; CVD mortality: METS-IR [16.7, 33.2] vs METS-IR [38.0, 77], HR, 1.14; 95% CI, 0.83–1.57).

**Table 3:** Multivariate Cox proportional-hazards regression models for cancer mortality and CVD mortality according to METS-IR tertiles in subjects older than 60 years.

		Cancer mortality				CVD mortality			
		Group 1	Group 2	Group 3	*p* for trend	Group 1	Group 2	Group 3	*p* for trend
		T1 [16.7, 33.2] (n = 10,055)	T2 [33.2, 38.0] (n = 10,054)	T3 [38.0, 77] (n = 10,055)		T1 [16.7, 33.2] (n = 10,055)	T2 [33.2, 38.0] (n = 10,054)	T3 [38.0, 77] (n = 10,055)	
New cases of death, n		382	390	463		158	159	197	
Mean follow-up, years		11.59	11.75	11.73		11.59	11.75	11.73	
Pearson-years of follow-up		116,497	118,171	117,942		116,497	118,172	117,943	
Incidence rate/1000 person -years		3.28	3.30	3.93		1.36	1.35	1.67	
Model 1	HR (95% CI)	1.00 (reference)	1.00 (0.87-1.15)	1.19 (1.04-1.36)	0.011	1.00 (reference)	0.98 (0.78-1.22)	1.22 (0.99-1.50)	0.057
	*p* value	–	0.979	0.012		–	0.844	0.063	
Model 2	HR (95% CI)	1.00 (reference)	1.09 (0.93-1.28)	1.39 (1.13-1.71)	0.001	1.00 (reference)	1.04 (0.81-1.34)	1.39 (1.01-1.90)	0.037
	*p* value	–	0.288	0.002		–	0.743	0.041	
Model 3	HR (95% CI)	1.00 (reference)	1.10 (0.94-1.29)	1.38 (1.12-1.69)	0.002	1.00 (reference)	1.03 (0.80-1.32)	1.30 (0.95-1.78)	0.097
	*p* value	–	0.244	0.002		–	0.844	0.103	
Model 4	HR (95% CI)	1.00 (reference)	1.06 (0.91-1.25)	1.23 (1.01-1.51)	0.048	1.00 (reference)	0.99 (0.77-1.27)	1.14 (0.83-1.57)	0.418
	*p* value	–	0.45	0.045		–	0.921	0.429	

Model 1: Unadjusted.

Model 2: adjusted for age, sex and WC.

Model 3: adjusted for age, sex, WC, SBP, DBP, ALT, AST, creatinine and LDL.

Model 4: adjusted for age, sex, WC, SBP, DBP, ALT, AST, creatinine, LDL, smoke, drink, exercise, HTN and DM.

## Discussion

In a community-based population of Korean adults aged over 60 years, our research has substantiated a significant positive association between METS-IR and all-cause mortality, even after adjusting for confounding factors. Additionally, when conducting a cause-specific analysis, METS-IR demonstrated a notable positive correlation with cancer-related mortality, while no statistically significant results were observed concerning CVD-related mortality.

Defining the older adults is a challenging task, considering the various biological, demographic, and sociological perspectives. However, it is a common practice to define the older population as individuals aged 60 or 65 and older for statistical and administrative purposes (32, 33). A previous study, aimed at investigating the relationship between changes in plasma proteomics across the lifespan and the occurrence of diseases, discovered a noteworthy trend. It revealed that among individuals aged 60 and older, there was a substantial enrichment of proteins associated with CVD. This finding strongly suggests an increased likelihood of CVD incidence in this age group (34). In this context, it is essential to establish correlation metrics for major diseases like CVD and associated mortality in the population 60 years and older.

Insulin plays a crucial role in maintaining glucose homeostasis primarily through promoting glucose uptake and exerts diverse effects on systemic target cells like muscle, adipose tissue and liver (9). When IR occurs, compensatory hyperinsulinemia develops to maintain normal glucose levels (35). IR can contribute to the development of atherosclerosis and the advancement of arterial plaque (36). Disturbed insulin signaling within the cells lining the innermost layer of blood vessels, which play a role in atherosclerosis, including endothelial cells, vascular smooth muscle cells, and macrophages, is thought to be a contributing factor. In other words, the original role of insulin in the endothelial cells, which is to counteract atherosclerosis, becomes impaired under conditions of IR, potentially accelerating the progression of atherosclerosis (37). Indeed, Prior investigations have demonstrated a correlation between IR and an elevated likelihood of CVD (17, 38, 39). The association between IR and cancer has also sparked widespread interest, leading to extensive research. It has been suggested that IR may contribute to an increased risk of cancer development (40–42). Tumorigenesis can be caused by the hallmark of IR, hyperinsulinemia, and the role of the insulin/insulin-like growth factor system associated with it (43).

Given these points, it can be inferred that higher IR may lead to increased mortality related to cancer and CVD. Some studies have yielded diverse results regarding the correlation between IR and mortality, encompassing both all-cause and cause-specific outcomes (27, 44–53). When interpreting our results within the context of previous research, both concordant and discordant findings emerge. In the analysis of the association between IR and cause-specific mortality, no study demonstrated a significant association with both cancer-related and CVD-related mortality. Some studies established an association between IR and all-cause mortality (44, 45), along with either CVD (27, 46–49) or cancer-related mortality (50, 51), while others failed to find associations with mortality (52, 53). This implies that understanding the shared pathophysiology, such as insulin resistance, between CVD and cancer, while simultaneously comprehending the distinct characteristics of each disease, is crucial for formulating tailored strategies for each. CVD and cancer are complex, multifactorial conditions, not attributable to a single cause (54). In some aspects, even the outcomes of shared pathways may differ between the occurrence and management of CVD and cancer. Angiogenesis is recognized as a driving force in cancer, characterized by pathological neovascularization. Therapeutic strategies aim to inhibit angiogenesis. Conversely, in ischemic diseases, angiogenesis is considered a therapeutic potential. In this context, insulin-like growth factor binding proteins (IGFBPs), a group of seven proteins essential for the transportation of insulin-like growth factor, play a significant role in angiogenesis. Each member within the IGFBP family exhibits varying effects on angiogenesis. IGFBP-1 and IGFBP-2 demonstrate pro-angiogenic effects, while IGFBP-4 and IGFBP-5 primarily exhibit anti-angiogenic properties. IGFBP-3 and IGFBP-7, depending on the cellular environment, possess dual characteristics, showing both pro- and anti-angiogenic effects (55).

Considering the reasons for inconsistent findings among previous studies, including our research results, several factors come to mind. Firstly, each study targeted a diverse study population with variations in age, sex, race, and other characteristics, and there were differences in sample sizes. Secondly, the variation in IR assessment methods across studies also contributes to these disparities. We utilized METS-IR as our IR marker, which is a variable with multiple components. Previous studies have used traditional IR marker like homeostatic model sssessment for insulin resistance (HOMA-IR) (45, 48–51), as well as non-insulin-based IR markers like the triglyceride glucose index (52). The former may face challenges in large-scale studies due to procedural and cost issues, while the latter lacks the inclusion of nutritional factors. METS-IR was able to overcome these limitations and provide more support in predicting cardiovascular metabolic diseases by integrating BMI (17). Moreover, In a previous study utilizing factor analysis, results similar to our study’s conclusions were derived. They formed a cluster of IR-related biomarkers, including higher BMI, FPG, TG, uric acid, and GGT activity and lower HDL-C levels. This cluster predicted cancer mortality, while HOMA-IR and fasting insulin failed to do so (56). The use of a multivariable marker, including BMI, to represent IR is similar between this study and our research. This suggests that multivariable markers, such as METS-IR, can be effective in predicting cancer-related mortality.

We conducted research beyond the initially studied population of 60 years and older to examine whether there are differences in the association between METS-IR and mortality based on age, specifically for those aged 65 and older, and those aged 40 to less than 60 (**Supplementary Tables S1–S4**). The findings indicate that METS-IR did not demonstrate significant associations with all-cause mortality and cause-specific mortality in both the 40 to less than 60 age group and the 65 and older age group. Reflecting on the reasons for this, in the 65 and older age group, the limited sample size appears to be a contributing factor. Additionally, for the 40 to 59 age group, the relatively low mortality rate and the distinctive characteristics of this age group, which focuses on a comparatively younger demographic, may have hindered the attainment of statistically significant results due to an insufficient follow-up duration to observe mortality occurrences.

On the other hand, there are still many aspects to address behind the potential use of METS-IR as an indicator for predicting all-cause mortality, particularly cancer-related mortality, in individuals aged 60 and above. In our study, we did not differentiate cancer types, and information regarding incidence rates was unavailable. Additional research on cancer types holds the potential to provide information on specific cancers that METS-IR may reflect, especially considering METS-IR’s inclusion of BMI as a marker. This suggests the possibility of obtaining insights into obesity-related cancers. Furthermore, additional studies that include the timing of occurrence can shed light on the possibility of being diagnosed with rapidly progressing cancers in terms of cancer-related mortality. Additionally, these studies may reveal the importance of internal factors, such as IR, in leading to mortality, even in the absence of differences in cancer types. Furthermore, prior research has suggested that genetic variations in insulin receptor genes can elevate the risk of obesity-related cancers (57). Considering this, by confirming cancers predominantly predicted by METS-IR and conducting additional studies to identify genetic variations associated with these cancers, we can establish the foundation for personalized medicine. This involves utilizing METS-IR to assess and manage individuals with such genetic variations.

Our study has both strengths and limitations. One key strength lies in its large, population-based cohort design, with a relatively higher number of mortality cases compared to other studies. Conversely, limitations include the fact that we focused solely on Korean adults aged 40 and older, limiting the generalizability of our findings to other countries, all age groups, and ethnic groups. Additionally, the information on the incidence rates of CVD and cancer is insufficient, and the study outcomes are restricted to mortality rates. Evaluation based on the types of cancer was not conducted. Finally, It is also possible that there are other residual confounding factors that were not adequately controlled for.

## Conclusions

METS-IR demonstrated a positive correlation with both all-cause and cancer-related mortality, making it a reliable predictor of mortality in individuals over 60 years old. These results highlight a previously unrecognized subgroup of elderly individuals at a significantly heightened risk of cancer-specific mortality. Early detection and intervention strategies could potentially benefit this identified group.

## Data availability statement

Publicly available datasets were analyzed in this study. This data can be found here: https://nih.go.kr/ko/main/contents.do?menuNo=300563.

## Ethics statement

The studies involving humans were approved by Yongin Severance Hospital (IRB number: 9-2023-0018). The studies were conducted in accordance with the local legislation and institutional requirements. The participants provided their written informed consent to participate in this study.

## Author contributions

HR: Conceptualization, Writing – original draft, Writing – review & editing. DJ: Conceptualization, Writing – original draft, Writing – review & editing. SH: Methodology, Writing – original draft, Writing – review & editing. BP: Methodology, Writing – original draft. YL: Funding acquisition, Supervision, Writing – review & editing.
