# Supplementary material for: METS-IR and all-cause mortality in Korean over 60 years old: Korean genome and epidemiology study-health examinees (KoGES-HEXA) cohorts
Source: Front Endocrinol (Lausanne). 2024 Mar 20;15:1346158. doi: 10.3389/fendo.2024.1346158 (PMC10987815; doi:10.3389/fendo.2024.1346158)
Supplement: Supplementary file 1 [file Table_1.docx]

**TABLE S1.** Hazard ratios and 95% confidence intervals for All-cause mortality according to METS-IR tertiles in subject 40-59 years.

|  | | Group 1 | Group 2 | | Group 3 | | *p* for trend |  |
| --- | --- | --- | --- | --- | --- | --- | --- | --- |
|  |  | T1 [16.7, 33.2] (n = 10,055) | T2 [33.2, 38.0] (n = 10,054) | | T3 [38.0, 77] (n = 10,055) | |  |  |
| New cases of death, n | | 512 | 587 | | 815 | |  |  |
| Mean follow-up, years | | 11.94 | 12.09 | | 11.98 | |  |  |
| Pearson-years of follow-up | | 368,084 | 372,641 | | 369,185 | |  |  |
| Incidence rate/1000 person -years | | 1.39 | 1.58 | | 2.21 | |  |  |
| Model 1 | HR (95% CI) | 1.00 (reference) | 1.13 (1.00-1.27) | | 1.05 (0.96-1.15) | | <0.001 |  |
|  | *p* value | - | 0.050 | | <0.001 | |  |  |
| Model 2 | HR (95% CI) | 1.00 (reference) | 0.94 (0.82-1.08) | | 1.21 (1.02-1.44) | | 0.020 |  |
|  | *p* value | - | 0.365 | | 0.031 | |  |  |
| Model 3 | HR (95% CI) | 1.00 (reference) | 0.96 (0.83-1.09) | | 1.20 (1.01-1.43) | | 0.030 |  |
|  | *p* value | - | 0.510 | | 0.041 | |  |  |
| Model 4 | HR (95% CI) | 1.00 (reference) | 0.92 (0.80-1.05) | | 1.03 (0.86-1.23) | | 0.690 |  |
|  | *p* value | - | 0.219 | | 0.743 | |  |  |
| Model 1: Unadjusted | | | |  | |  |  |  |
| Model 2: adjusted for age, sex, and WC | | | | | | |  |  |
| Model 3: adjusted for age, sex, WC, SBP, DBP, ALT, AST, creatinine and LDL | | | | | | | | |
| Model 4: adjusted for age, sex, WC, SBP, DBP, ALT, AST, creatinine, LDL, smoke, drink, exercise, HTN, and DM | | | | | | | | |

**TABLE S2.** Hazard ratios and 95% confidence intervals for All-cause mortality according to METS-IR tertiles in subject older than 65 years.

|  | | Group 1 | Group 2 | | Group 3 | | *p* for trend |  |
| --- | --- | --- | --- | --- | --- | --- | --- | --- |
|  |  | T1 [16.7, 33.2] (n = 10,055) | T2 [33.2, 38.0] (n = 10,054) | | T3 [38.0, 77] (n = 10,055) | |  |  |
| New cases of death, n | | 592 | 563 | | 587 | |  |  |
| Mean follow-up, years | | 11.50 | 11.7 | | 11.6 | |  |  |
| Pearson-years of follow-up | | 49,837 | 50,545 | | 50,435 | |  |  |
| Incidence rate/1000 person -years | | 11.90 | 11.10 | | 11.60 | |  |  |
| Model 1 | HR (95% CI) | 1.00 (reference) | 0.93 (0.83-1.04) | | 0.97 (0.87-1.09) | | 0.635 |  |
|  | *p* value | - | 0.201 | | 0.634 | |  |  |
| Model 2 | HR (95% CI) | 1.00 (reference) | 1.11 (0.97-1.27) | | 1.41 (1.18-1.67) | | <0.001 |  |
|  | *p* value | - | 0.123 | | <0.001 | |  |  |
| Model 3 | HR (95% CI) | 1.00 (reference) | 1.09 (0.95-1.24) | | 1.32 (1.11-1.57) | | 0.002 |  |
|  | *p* value | - | 0.232 | | 0.002 | |  |  |
| Model 4 | HR (95% CI) | 1.00 (reference) | 1.04 (0.91-1.19) | | 1.14 (0.96-1.36) | | 0.139 |  |
|  | *p* value | - | 0.593 | | 0.139 | |  |  |
| Model 1: Unadjusted | | | |  | |  |  |  |
| Model 2: adjusted for age, sex, and WC | | | | | | |  |  |
| Model 3: adjusted for age, sex, WC, SBP, DBP, ALT, AST, creatinine and LDL | | | | | | | | |
| Model 4: adjusted for age, sex, WC, SBP, DBP, ALT, AST, creatinine, LDL, smoke, drink, exercise, HTN, and DM | | | | | | | | |

**TABLE S3.** Multivariate Cox proportional-hazards regression models for cancer mortality and CVD mortality according to METS-IR tertiles in subject 40-59 years

|  | | Cancer mortality | | | | CVD mortality | | | |
| --- | --- | --- | --- | --- | --- | --- | --- | --- | --- |
|  |  | Group 1 | Group 2 | Group 3 | *p* for trend | Group 1 | Group 2 | Group 3 | *p* for trend |
|  |  | T1 [16.7, 33.2] (n = 10,055) | T2 [33.2, 38.0] (n = 10,054) | T3 [38.0, 77] (n = 10,055) |  | T1 [16.7, 33.2] (n = 10,055) | T2 [33.2, 38.0] (n = 10,054) | T3 [38.0, 77] (n = 10,055) |  |
| New cases of death, n | | 275 | 317 | 399 |  | 50 | 76 | 130 |  |
| Mean follow-up, years | | 11.94 | 12.09 | 11.98 |  | 11.94 | 12.09 | 11.98 |  |
| Pearson-years of follow-up | | 368,084 | 372,641 | 369,185 |  | 368,084 | 372,641 | 369,185 |  |
| Incidence rate/1000 person -years | | 0.75 | 0.85 | 1.08 |  | 0.14 | 0.20 | 0.35 |  |
| Model 1 | HR (95% CI) | 1.00 (reference) | 1.13 (0.96-1.33) | 1.45 (1.24-1.69) | <0.001 | 1.00 (reference) | 1.50 (1.05-2.14) | 2.59 (1.87-3.59) | <0.001 |
|  | *p* value | - | 0.134 | <0.001 |  | - | 0.027 | <0.001 |  |
| Model 2 | HR (95% CI) | 1.00 (reference) | 0.97 (0.81-1.17) | 1.17 (0.92-1.49) | 0.185 | 1.00 (reference) | 1.25 (0.84-1.86) | 2.03 (1.25-3.30) | 0.003 |
|  | *p* value | - | 0.767 | 0.202 |  | - | 0.271 | 0.004 |  |
| Model 3 | HR (95% CI) | 1.00 (reference) | 0.99 (0.83-1.19) | 1.16 (0.91-1.48) | 0.213 | 1.00 (reference) | 1.23 (0.83-1.84) | 1.97 (1.21-3.20) | 0.004 |
|  | *p* value | - | 0.937 | 0.225 |  | - | 0.299 | 0.006 |  |
| Model 4 | HR (95% CI) | 1.00 (reference) | 0.96 (0.80-1.16) | 1.04 (0.82-1.34) | 0.717 | 1.00 (reference) | 1.17 (0.79-1.74) | 1.59 (0.97-2.59) | 0.054 |
|  | *p* value | - | 0.695 | 0.728 |  | - | 0.438 | 0.064 |  |
| Model 1: Unadjusted | | |  |  |  |  |  |  |  |
| Model 2: adjusted for age, sex and WC | | | | |  |  |  |  |  |
| Model 3: adjusted for age, sex, WC, SBP, DBP, ALT, AST, creatinine and LDL | | | | | | | | | |
| Model 4: adjusted for age, sex, WC, SBP, DBP, ALT, AST, creatinine, LDL, smoke, drink, exercise, HTN and DM | | | | | | |  |  |  |

**TABLE S4.** Multivariate Cox proportional-hazards regression models for cancer mortality and CVD mortality according to METS-IR tertiles in subject older than 65 years

|  | | Cancer mortality | | | | CVD mortality | | | |
| --- | --- | --- | --- | --- | --- | --- | --- | --- | --- |
|  |  | Group 1 | Group 2 | Group 3 | *p* for trend | Group 1 | Group 2 | Group 3 | *p* for trend |
|  |  | T1 [16.7, 33.2] (n = 10,055) | T2 [33.2, 38.0] (n = 10,054) | T3 [38.0, 77] (n = 10,055) |  | T1 [16.7, 33.2] (n = 10,055) | T2 [33.2, 38.0] (n = 10,054) | T3 [38.0, 77] (n = 10,055) |  |
| New cases of death, n | | 227 | 227 | 259 |  | 105 | 104 | 121 |  |
| Mean follow-up, years | | 11.50 | 11.7 | 11.6 |  | 11.50 | 11.7 | 11.6 |  |
| Pearson-years of follow-up | | 49,837 | 50,545 | 50,435 |  | 49,837 | 50,545 | 50,435 |  |
| Incidence rate/1000 person -years | | 4.55 | 4.49 | 5.14 |  | 2.11 | 2.06 | 2.40 |  |
| Model 1 | HR (95% CI) | 1.00 (reference) | 0.98 (0.81-1.18) | 1.12 (0.94-1.34) | 0.201 | 1.00 (reference) | 0.96 (0.73-1.26) | 1.13 (0.87-1.47) | 0.353 |
|  | *p* value | - | 0.825 | 0.209 |  | - | 0.784 | 0.364 |  |
| Model 2 | HR (95% CI) | 1.00 (reference) | 1.10 (0.89-1.36) | 1.39 (1.06-1.83) | 0.015 | 1.00 (reference) | 1.10 (0.80-1.50) | 1.50 (1.01-2.23) | 0.042 |
|  | *p* value | - | 0.396 | 0.016 |  | - | 0.562 | 0.045 |  |
| Model 3 | HR (95% CI) | 1.00 (reference) | 1.09 (0.89-1.35) | 1.36 (1.04-1.78) | 0.025 | 1.00 (reference) | 1.06 (0.78-1.45) | 1.39 (0.94-2.07) | 0.097 |
|  | *p* value | - | 0.402 | 0.026 |  | - | 0.698 | 0.100 |  |
| Model 4 | HR (95% CI) | 1.00 (reference) | 1.06 (0.86-1.32) | 1.21 (0.92-1.59) | 0.176 | 1.00 (reference) | 1.02 (0.75-1.39) | 1.19 (0.80-1.78) | 0.389 |
|  | *p* value | - | 0.564 | 0.178 |  | - | 0.907 | 0.394 |  |
| Model 1: Unadjusted | | |  |  |  |  |  |  |  |
| Model 2: adjusted for age, sex and WC | | | | |  |  |  |  |  |
| Model 3: adjusted for age, sex, WC, SBP, DBP, ALT, AST, creatinine and LDL | | | | | | | | | |
| Model 4: adjusted for age, sex, WC, SBP, DBP, ALT, AST, creatinine, LDL, smoke, drink, exercise, HTN and DM | | | | | | |  |  |  |

**TABLE S5.** Imputation analysis

|  |  | All-cause mortality | | | | Cancer mortality | | | | CVD mortality | | | |
| --- | --- | --- | --- | --- | --- | --- | --- | --- | --- | --- | --- | --- | --- |
|  | | Group 1 | Group 2 | Group 3 | *p* for trend | Group 1 | Group 2 | Group 3 | *p* for trend | Group 1 | Group 2 | Group 3 | *p* for trend |
| Model 1 | HR (95% CI) | 1.00 (reference) | 0.94 (0.86-1.03) | 1.06 (0.97-1.15) | 0.218 | 1.00 (reference) | 0.98 (0.85-1.12) | 1.19 (1.04-1.36) | 0.009 | 1.00 (reference) | 0.96 (0.78-1.19) | 1.19 (0.97-1.46) | 0.082 |
|  | *p* value | - | 0.168 | 0.230 |  | - | 0.757 | 0.011 |  | - | 0.722 | 0.090 |  |
| Model 2 | HR (95% CI) | 1.00 (reference) | 1.11 (1.00-1.24) | 1.46 (1.28-1.67) | <0.001 | 1.00 (reference) | 1.09 (0.93-1.28) | 1.45 (1.19-1.77) | <0.001 | 1.00 (reference) | 1.05 (0.82-1.34) | 1.42 (1.04-1.92) | 0.023 |
|  | *p* value | - | 0.041 | <0.00.1 |  | - | 0.285 | <0.00.1 |  | - | 0.713 | 0.026 |  |
| Model 3 | HR (95% CI) | 1.00 (reference) | 1.10 (0.99-1.22) | 1.38 (1.21-1.58) | <0.001 | 1.00 (reference) | 1.10 (0.94-1.29) | 1.43 (1.17-1.74) | <0.001 | 1.00 (reference) | 1.02 (0.80-1.31) | 1.32 (0.97-1.79) | 0.071 |
|  | *p* value | - | 0.075 | <0.001 |  | - | 0.255 | <0.001 |  | - | 0.844 | 0.076 |  |
| Model 4 | HR (95% CI) | 1.00 (reference) | 1.05 (0.95-1.16) | 1.21 (1.06-1.38) | 0.005 | 1.00 (reference) | 1.06 (0.90-1.24) | 1.27 (1.04-1.55) | 0.018 | 1.00 (reference) | 0.98 (0.77-1.25) | 1.15 (0.84-1.57) | 0.367 |
|  | *p* value | - | 0.357 | 0.005 |  | - | 0.476 | 0.020 |  | - | 0.878 | 0.380 |  |
| Model 1: Unadjusted | | | | | | | | | | | | | |
| Model 2: adjusted for age, sex, and WC. | | | | | | | | | | | | | |
| Model 3: adjusted for age, sex, WC, SBP, DBP, ALT, AST, LDL, and creatinine | | | | | | | | | | | | | |
| Model 4: adjusted for age, sex, WC, SBP, DBP, ALT, AST, creatinine, LDL, smoke, drink, exercise, HTN, and DM | | | | | | | | | | | | | |
